# The road most travelled: the geographic distribution of road traffic injuries in England

**DOI:** 10.1186/1476-072X-12-30

**Published:** 2013-06-05

**Authors:** Rebecca Steinbach, Phil Edwards, Chris Grundy

**Affiliations:** 1 Department of Social and Environmental Health, London School of Hygiene and Tropical Medicine, 15-17 Tavistock Place, London WC1H 9SH, UK; 2 Department of Population Health, London School of Hygiene and Tropical Medicine, Keppel Street, London WC1E 7HT, UK

**Keywords:** Accidents, Wounds and injuries, Social differences

## Abstract

**Background:**

Both road safety campaigns and epidemiological research into social differences in road traffic injury risk often assume that road traffic injuries occur close to home. While previous work has examined distance from home to site of collision for child pedestrians in local areas, less is known about the geographic distribution of road traffic injuries from other modes. This study explores the distribution of the distance between home residence and collision site (crash distance) by mode of transport, geographic area, and social characteristics in England.

**Methods:**

Using 10 years of road casualty data collected by the police, we examined the distribution of crash distance by age, sex, injury severity, area deprivation, urban/rural status, year, day of week, and, in London only, ethnic group.

**Results:**

54% of pedestrians, 39% of cyclists, 17% of powered two-wheeler riders and 16% of car occupants were injured within 1 km of home. 82% of pedestrians, 83% of cyclists, 54% of powered two-wheeler and 53% of car occupants were injured within 5 km of home. We found some social and geographic differences in crash distance: for all transport modes injuries tended to occur closer to home in more deprived or urban areas; younger and older pedestrians and cyclists were also injured closer to home. Crash distance appears to have increased over time for pedestrian, cyclist and car occupant injuries, but has decreased over time for powered two-wheeler injuries.

**Conclusions:**

Injuries from all travel modes tend to occur quite close to home, supporting assumptions made in epidemiological and road safety education literature. However, the trend for increasing crash distance and the social differences identified may have methodological implications for future epidemiological studies on social differences in injury risk.

## Background

While a growing body of work examines social differences in road traffic injury, there has been relatively little work exploring the geographic distribution of injuries [1], in particular the distribution of distance from home. A number of road safety initiatives have launched campaigns on the assumption that road traffic collisions occur close to home. In 2003, the Department for Transport’s THINK campaign launched a “Knowing the Road” commercial as part of their Hedgehogs children’s road safety advertising videos, which addresses awareness of dangers on roads close to home [2]. More recently, in 2006 Transport for London’s ‘Losing Control’ television and cinema advertising campaign warned motorcyclists to “Ride the roads you know as carefully as those you don't” [3].

The few studies that have examined distance from home to site of road traffic collision (which we will refer to as crash distance) focus on small areas and restrict analyses to pedestrians or children. Some international evidence using data from one major trauma centre in the US, suggests that children and older citizens tend to be injured as pedestrians closer to home compared to other adults, and more severe pedestrian injuries occur further from home compared to less severe injuries [4], but internationally there is little research on crash distances for other modes. Within the UK, examination of crash distance has focused on children [5], and child pedestrians in particular [6-8], mainly for methodological reasons.

In addition to a comparatively poor child pedestrian injury record overall in the UK [9], there are well reported inequalities in child pedestrian injury risk. Research has documented inequalities in injury risk by employment status [10], area deprivation [11-13] and ethnicity [14-16]. Methodologically, in order to (a) maximize usable data (as home location is often missing from data) and (b) find appropriate denominators for injury rates, these studies often assume that child pedestrian injuries occur close to home. A study on child fatalities in the Northern region of England found that 80% of child pedestrian injuries occurred within 1.6 km of home [8], a finding replicated in a study focusing on the city of Salford [5]. A more recent study from London found that on average children were injured 1.7 km from home [7]. There is less evidence on whether distance varies by social characteristics, an important issue for studies that examine social differences in risk. A few of these small area studies have examined crash distance by age group and have found that distance was shorter among younger children [5-7], however there is a paucity of studies that examine crash distance by deprivation and ethnicity.

## Methods

We obtained 10 years (2000–2009) of Police STATS19 data, the official data set of all injuries that occur on public highways in the UK from the Department for Transport (DfT). Officers collect data on the easting and northing coordinates of each collision location and the postcode of residence of each injured person. The DfT supplied us with straight line ‘crow flies’ distances from the site of collision to the centroid of the postcode of residence. Data also include age of casualty, which we grouped into five year age bands for analysis, sex, mode of travel (pedestrian, cyclists, powered two-wheeler, or car occupant), severity of injury (fatal, serious or slight injury), the government office region where the collision occurred, rural or urban status, and the Index of Multiple Deprivation (IMD) score of the Lower Super Output Area (LSOA) of the casualty’s residence. For analysis, all LSOAs in England were ranked according to IMD score and grouped into deciles (1 least deprived to 10 most deprived). Analyses also consider trends in crash distance by year and day of week.

Nationally, police do not collect data on ethnicity of casualties, however in London ethnicity has been collected since 1996. To explore ethnic differences in distance we obtained 10 years of data (2000–2009) from Transport for London’s London Road Safety Unit. The measure of ethnicity used is the six-category Police National Computer ‘Identity Code’, which we grouped into three broad categories based on previous research [16] ‘White’ (white-skinned European, dark-skinned European); ‘Black’ (Afro-Carribean); and ‘Asian’ (Asian). We calculated distance in the same manner as the DfT, a straight line ‘crow flies’ distance from the centroid of each casualty’s postcode of residence to the coordinates of the site of collision. We focus our analysis on child pedestrians in London due to identified social inequalities in risk in the literature [16]. Our data on child pedestrian injury in London also included information on time of road traffic collision. We have included an analysis of crash distance by time of day grouped into 5 categories (10pm-7am, 7am-9am, 9am-3pm, 3pm-6pm, and 6pm-10pm) during weekdays.

### Analysis

We calculated the median crash distance with interquartile ranges (25th percentile to 75th percentile) by travel mode in each population subgroup. To statistically compare subgroups we evaluated the difference in means of log- transformed variables using analysis of variance (ANOVA).

## Results

Between 2000–2009, 2,430,542 injuries were reported in STATS19 in England. Of those injuries 12% occurred to pedestrians, 7% to cyclists, 10% to powered two wheeler riders, 63% to car occupants and 8% to travellers using other transport modes (e.g. bus occupants, goods vehicle occupants, agricultural vehicle occupants). 1,617,482 (67%) had valid information on postcode of residence and therefore information on crash distance. Median distance was longest for car occupant injuries (4.5 km, interquartile [IQR] 1.7-12.2) followed by powered two wheeler injuries (4.3 km, IQR 1.6-10.8) and was shorter for cyclist injuries (1.5 km, IQR 0.6-3.5) and pedestrian injuries (0.8, IQR 0.2-3.2)].

**Figure 1:**
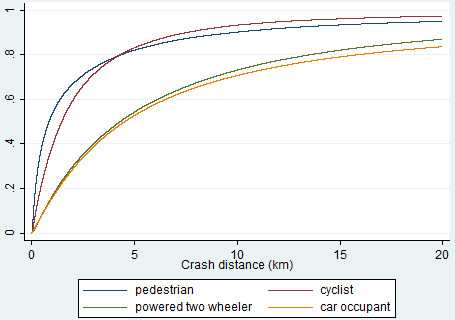
Cumulative distribution of crash distance by travel mode.

Figure 1 shows the cumulative distribution of crash distance by mode of travel. The majority of injuries in all travel modes occurs relatively close to home, though the distribution varies by mode (p = 0.001) with pedestrians and cyclists injured closer to home than to powered two-wheeler riders and car occupants.

54% of pedestrians, 39% of cyclists, 17% of powered two-wheeler occupants and 16% of car occupants were injured within 1 km of home. 82% of pedestrians, 83% of cyclists, 54% of powered two-wheeler and 53% of car occupants were injured within 5 km of home.

**Figure 2:**
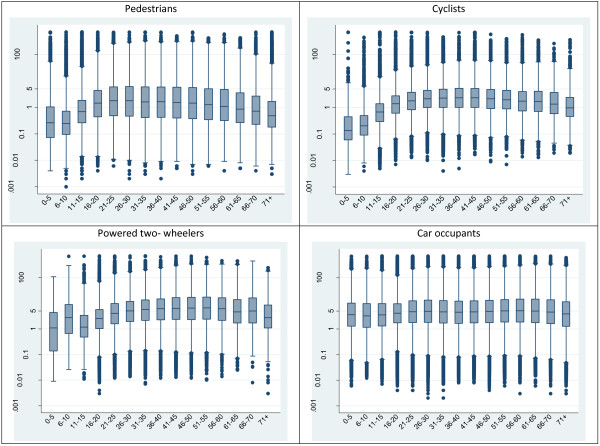
Distribution of crash distance by travel mode and age group.

Younger and older pedestrians and cyclists tended to be injured closer to home than adult age groups (Figure 2). Powered- two wheeler riders show a similar relationship between age and crash distance though numbers of powered two wheeler injuries in young age groups are very small (Additional file 1). Median crash distance for car occupants was longest in those between the ages of 51–65 and shortest among those under 15. There was evidence for differences in crash distance by age for all travel modes (p < 0.001).

Median crash distance in men was longer than in women for all travel modes, although absolute differences in distance tended to be relatively small (Additional file 1).

Fatal injuries tended to occur further from home for all travel modes (Additional file 1), except in pedestrians where slight injuries (median distance 0.86 km, IQR 0.25-3.22) occurred similarly close to home compared to fatal injuries (median distance 0.84 km, IQR 0.24-3.75), and further from home than serious injuries (median distance 0.77 km, IQR 0.22-3.05).

**Figure 3:**
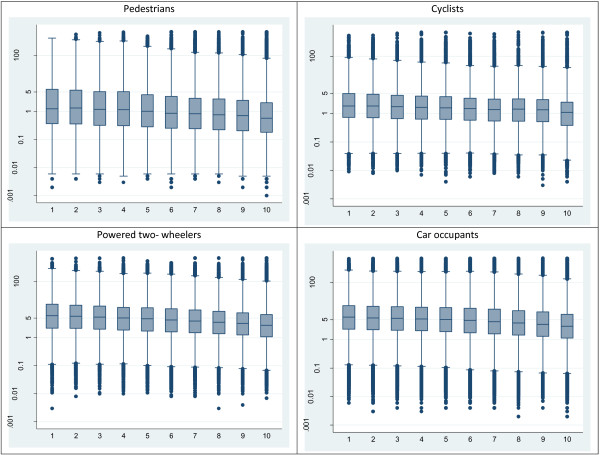
Distribution of crash distance by travel mode and decile of IMD.

For all travel modes, injuries tended to occur closer to home in more deprived areas compared to relatively affluent areas (Figure 3). There was evidence for differences in crash distance by decile of IMD for all travel modes (p < 0.001).

Injuries in rural areas occurred further from home than injuries in urban areas (Additional file 1). This was particularly true for car occupants where median distance in rural areas (8.26 km, IQR 3.43-21.70) was nearly three times longer than distance in urban areas (2.80 km, IQR 1.09-6.92). There was evidence for differences in crash distance by urban rural status for all travel modes (p < 0.001).

There was evidence for differences in distance by region: median distance for pedestrians, cyclists and powered-two wheeler riders were longest in London (1.31 km, IQR 0.34-4.94; 2.46 km, IQR 0.99-5.19; 5.05 km, IQR 2.04-10.94), while distance for car occupants was shortest in London (3.58km, IQR 1.39-8.38). Distance for pedestrians was shortest in the North East (0.67 km, IQR 0.18-2.55) and the North West (0.67 km, IQR 0.20-2.45). Distance for cyclists was shortest in the North East (1.07 km, IQR 0.33-2.95), while distance for powered-two wheeler riders was shortest in the West Midlands (3.50 km, IQR 1.34-8.68). Distance for car occupants was longest in the East of England (6.04 km, IQR 2.06 – 16.80). There was evidence for difference in crash distance by region for all travel modes (p < 0.001).

Crash distance appears to be increasing over time for pedestrians, cyclists and car occupants but appears to decrease over time for powered two-wheeler riders (Additional file 1). There was evidence for a difference in distance by year for all travel modes (p < 0.001).

Fewer casualties of all types occur on Sundays compared to other days of the week, but those that occur were further from home for car occupants and powered two-wheeler riders. Pedestrian injuries occur furthest from home on Saturday and Sundays, while cycling casualties occur closest to home on Saturdays and Sundays. There was evidence for a difference in crash distance by day of the week for all travel modes (p < 0.001).

### Child pedestrians in London

Between 2000–2009 there were 15,508 children aged 0–15 injured as pedestrians on London’s road. Ethnicity was coded for 85% of the data. There were 6,971 ‘White’ child pedestrian injuries (45%), 4,043 ‘Black’ child pedestrian injuries (26%), and 1,816 ‘Asian’ child pedestrian injuries (12%). 9,044 (58%) of the data had valid postcodes of residence, enabling us to calculate crash distance.

The median crash distance was 0.67 km (IQR 0.20-2.12) among children injured as pedestrians on London’s roads. Older children [11-15] tended to be injured further away (0.96 km, IQR 0.32-2.52) than children in younger age groups (Table 1). Median crash distance among girls was significantly longer than boys (p < 0.001) though the actual difference in distance was around 100 metres. Slight injuries tended to occur further from home than fatal or serious injuries, however, analysis of variance found no evidence that the distances were different than each other by injury severity (p = 0.792). Crash distances tended to decrease with increasing levels of deprivation among child pedestrians in London. Median distance among children living in the most deprived areas of London (0.49 km, IQR 0.16-1.80) was half as long as median distance among children living in the most affluent areas of London (1.01 km, IQR 0.34-2.45). There was evidence of a difference in crash distance by IMD of residence for child pedestrians in London (p < 0.001). ‘Asian’ children were injured as pedestrians closer to home (0.48 km, IQR 0.13-1.69) than ‘White’ (0.67 km, IQR 0.20-2.04) or ‘Black’ children (0.71 km, IQR 0.23-2.45). There was evidence of a difference in crash distance by ethnicity for child pedestrians in London (p < 0.001).

**Table 1:** Median crash distance among child pedestrians in London

**Characteristic**	**n**	**25th**	**50th**	**75th**	P value*
**Age**					
0-5	1521	0.11	0.49	1.97	<0.0001
6-10	2545	0.12	0.38	1.31	
11-15	4978	0.32	0.96	2.52	
**Sex**					
Male	5323	0.17	0.62	2.08	<0.0001
Female	3721	0.23	0.73	2.18	
**Severity**					
Fatal	36	0.21	0.57	4.12	0.7915
Serious	1714	0.18	0.60	2.23	
Slight	7294	0.20	0.68	2.11	
**IMD of home residence**					
(least deprived) 1	443	0.34	1.01	2.45	<0.0001
2	542	0.24	0.73	2.28	
3	537	0.25	0.82	2.12	
4	677	0.22	0.64	1.89	
5	731	0.21	0.73	2.33	
6	963	0.19	0.68	2.17	
7	1030	0.17	0.57	1.84	
8	1197	0.18	0.59	1.84	
9	1352	0.18	0.58	1.78	
(most deprived) 10	1382	0.16	0.49	1.80	
**Ethnic group**					
White	4140	0.20	0.67	2.04	<0.0001
Black	2378	0.23	0.71	2.45	
Asian	1029	0.13	0.48	1.69	
**Year**					
2000	1072	0.16	0.58	1.98	<0.0001
2001	1131	0.17	0.61	2.07	
2002	938	0.18	0.54	1.74	
2003	900	0.17	0.57	2.13	
2004	898	0.19	0.64	1.78	
2005	924	0.23	0.71	2.38	
2006	833	0.23	0.86	2.46	
2007	831	0.23	0.73	2.24	
2008	765	0.22	0.76	2.47	
2009	752	0.22	0.80	2.29	
**Day of week**					
Sunday	761	0.14	0.69	2.94	0.3077
Monday	1337	0.21	0.68	2.01	
Tuesday	1412	0.19	0.68	2.10	
Wednesday	1536	0.20	0.65	1.96	
Thursday	1432	0.21	0.62	1.93	
Friday	1510	0.21	0.68	1.97	
Saturday	1056	0.18	0.71	2.83	
**Time of day (weekdays only)**					
10pm - 7am	122	0.34	1.00	2.60	<0.0001
7am-9am	1277	0.25	0.64	1.78	
9am-3pm	1209	0.22	0.73	2.36	
3pm-6pm	3257	0.23	0.73	2.08	
6pm-10pm	1362	0.13	0.45	1.56	

*P value of ANOVA F-test.

Distance for child pedestrians in London is variable by year, but distances tend to be increasing over time. Analysis of variance found a significant difference in distance from home by year (p < 0.001). Distances appear to be relatively similar across all days of the week. Analysis of variance found a no difference in distance from home by day of week (p = 0.308). Child pedestrians appear to be injured closest to home between 6pm-10pm on weekdays (0.45 km, IQR 0.13-1.56), followed by the time of morning commute 7am-9am (0.64 km, IQR 0.25-1.78) while crash distance appears to be relatively similar during the time of school hours 9am-3pm (0.73 km, IQR 0.22-2.36) and during the time of the commute home from school 3pm-6pm (0.73 km, IQR 0.23-2.08). Analysis of variance found a significant difference in distance from home by time of day on weekdays (p < 0.001).

## Discussion

We examined distance from home to site of collision across England for all travel modes and found that injuries from all modes tend to occur quite close to home, confirming assumptions in the epidemiological and road safety education literature. Exposure is a likely mechanism to explain these findings. People tend to be injured close to home because that is where much of their transport activity takes place. Area familiarity may also play a role, as travellers develop expectations about the road environments which they encounter often. Indeed, evidence suggests that for drivers, eye movement changes after repeated exposure to a particular road environment, which may result in inadequate responses to unexpected changes in that environment [17]. While a growing body of work addresses the familiarity hypothesis among drivers [18,19], evidence is less clear for other types of road users [20,21].

Our analysis suggests that distances are increasing over time for pedestrians, cyclists, and car occupants. Over the same time period data from the National Travel Survey suggests that average distances travelled by walking and motorcycles were relatively stable, distances travelled by car decreased over time, while distances travelled by cycling increased [22]. We also found that car occupant, powered two-wheeler and pedestrian injuries occurred relatively far from home on Sundays suggesting that people travel further from home for leisure activities compared to their daily commutes.

Our findings on the relationship between age and distance are similar to previous international work on pedestrian injuries [4] and national work on child pedestrian injuries [5-7]. We also found other social differences in crash distances. Because we obtained a large amount of data, differences in crash distance from home by subgroup tended to be statistically significant, even if actual differences were quite small. But a few subgroup differences stand out: for all user modes, injuries tend to occur closer to home in more deprived and urban areas.

Within London, we found some social differences in distance among child pedestrians. ‘Asian’ children and children from deprived areas appear to be injured closer to home. These findings may have implications for studies examining social differences in risk. The methodological challenges of finding appropriate denominators in which to assess area-level risk are well known [23,24]. Research into social differences in pedestrian injury risk estimates injury rates by the ratio of the number of injuries that occur in an area (numerator) with the resident population (denominator). Other studies use an alternative estimate for the denominator and link injured child pedestrians to the areas in which they live. The most appropriate method is under debate [13,24], but our findings on social differences in crash distance suggest that some estimates of injury risk may be more accurate than others. Further work is needed to examine the methodological assumptions of studies addressing social differences in injury risk.

A limitation of our analysis is the under-reporting of road traffic injuries in the Stats19 data [25]. This under-reporting of injuries, however, will only affect our estimates if unreported injuries differ from reported injuries in terms of crash distance. A further threat is the 33% of reported injuries that are missing data on postcode of residence. Again, this missing data will affect our estimates if the distribution of crash distance differs among those who do and do not report a postcode of residence. Despite these weaknesses, we were able to examine over two million road traffic injuries to provide the most comprehensive description of distance from home to site of collision in England to date.

That our findings on pedestrian injury are similar to American findings suggests that our results may be generalisable to places with similar road environments and travel patterns. As there is good evidence that reducing speeds and (re)designing road environments for all types of road users are effective ways of reducing road traffic injuries [26-28], our findings may imply that these types of interventions are particularly important in residential areas in high income countries. However, more work looking at crash distance in low and middle income countries, where the burden of road traffic injury is highest [26], is needed.

## Competing interests

The authors declare that they have no competing interests.

## Authors’ contributions

RS, PE, and CG all contributed to the design of the study, analysis of the data, and writing of the manuscript. All authors read and approved the final manuscript.

## Supplementary Material

Additional file 1: Table A1 Median crash distance among pedestrians in England 2000-2009. **Table A2** Median crash distance among cyclists in England 2000-2009. **Table A3** Median crash distance among powered two-wheelers in England 2000-2009. **Table A4** Median crash distance among car occupants in England 2000-2009. Click here for file
