# Supplementary material for: The road most travelled: the geographic distribution of road traffic injuries in England
Source: Int J Health Geogr. 2013 Jun 5;12:30. doi: 10.1186/1476-072X-12-30 (PMC3680192; doi:10.1186/1476-072X-12-30)
Supplement: Additional file 1: Table A1 — Median crash distance among pedestrians in England 2000-2009. Table A2 Median crash distance among cyclists in England 2000-2009. Table A3 Median crash distance among powered two-wheelers in England 2000-2009. Table A4 Median crash distance among car occupants in England 2000-2009. [file 1476-072X-12-30-S1.docx]

Table A1 Median crash distance among pedestrians in England 2000-2009

| **Characteristic** | **n** | **25th** | **50th** | **75th** | **Pvalue*** |
| --- | --- | --- | --- | --- | --- |
| **Age** | | | | | |
| 0-5 | 9575 | 0.07 | 0.26 | 1.04 | <0.0001 |
| 6-10 | 18204 | 0.09 | 0.25 | 0.73 |  |
| 11-15 | 32168 | 0.26 | 0.69 | 1.84 |  |
| 16-20 | 19390 | 0.44 | 1.44 | 4.37 |  |
| 21-25 | 13317 | 0.48 | 1.80 | 5.96 |  |
| 26-30 | 10628 | 0.46 | 1.79 | 6.14 |  |
| 31-35 | 9201 | 0.42 | 1.62 | 6.00 |  |
| 36-40 | 8775 | 0.42 | 1.64 | 5.89 |  |
| 41-45 | 6069 | 0.39 | 1.51 | 5.78 |  |
| 46-50 | 7926 | 0.38 | 1.45 | 5.64 |  |
| 51-55 | 5618 | 0.34 | 1.28 | 5.06 |  |
| 56-60 | 5197 | 0.31 | 1.09 | 4.76 |  |
| 61-65 | 4344 | 0.26 | 0.86 | 3.56 |  |
| 66-70 | 4096 | 0.23 | 0.72 | 2.67 |  |
| 71+ | 14437 | 0.18 | 0.49 | 1.67 |  |
| **Sex** | | | | | |
| Male | 96764 | 0.24 | 0.89 | 3.47 | <0.0001 |
| Female | 74711 | 0.24 | 0.78 | 2.89 |  |
| **Severity** | | | | | |
| Fatal | 3035 | 0.24 | 0.84 | 3.75 | <0.0001 |
| Serious | 34839 | 0.22 | 0.77 | 3.05 |  |
| Slight | 133627 | 0.25 | 0.86 | 3.22 |  |
| **IMD of home residence** | | | | | |
| 1 | 8898 | 0.37 | 1.26 | 6.24 | <0.0001 |
| 2 | 10030 | 0.36 | 1.35 | 5.83 |  |
| 3 | 10799 | 0.33 | 1.20 | 5.16 |  |
| 4 | 11928 | 0.32 | 1.18 | 5.17 |  |
| 5 | 13597 | 0.28 | 1.03 | 4.02 |  |
| 6 | 15355 | 0.25 | 0.88 | 3.47 |  |
| 7 | 18223 | 0.24 | 0.83 | 3.01 |  |
| 8 | 22085 | 0.23 | 0.77 | 2.82 |  |
| 9 | 26821 | 0.21 | 0.72 | 2.54 |  |
| 10 | 33765 | 0.18 | 0.59 | 2.07 |  |
| **Urban/Rural status** | | | | | |
| Urban | 152957 | 0.24 | 0.83 | 3.00 | <0.0001 |
| Rural | 18473 | 0.27 | 1.01 | 5.69 |  |

| **Characteristic** | **n** | **25th** | **50th** | **75th** | **Pvalue*** |
| --- | --- | --- | --- | --- | --- |
| **Government Office Region** | | | | | |
| North East | 7761 | 0.18 | 0.67 | 2.55 | <0.0001 |
| North West | 29067 | 0.20 | 0.67 | 2.45 |  |
| Yorkshire and the Humber | 18907 | 0.22 | 0.76 | 2.88 |  |
| East Midlands | 9102 | 0.24 | 0.79 | 2.96 |  |
| West Midlands | 25425 | 0.21 | 0.71 | 2.47 |  |
| East of England | 13078 | 0.24 | 0.81 | 3.22 |  |
| London | 35549 | 0.34 | 1.31 | 4.94 |  |
| South East | 22513 | 0.26 | 0.88 | 3.25 |  |
| South West | 9900 | 0.26 | 0.88 | 3.35 |  |
| **Year** |  |  |  |  |  |
| 2000 | 15027 | 0.23 | 0.79 | 3.00 | <0.0001 |
| 2001 | 15446 | 0.22 | 0.77 | 2.96 |  |
| 2002 | 15643 | 0.24 | 0.79 | 2.98 |  |
| 2003 | 14954 | 0.23 | 0.81 | 3.12 |  |
| 2004 | 16530 | 0.23 | 0.81 | 3.15 |  |
| 2005 | 18714 | 0.25 | 0.87 | 3.29 |  |
| 2006 | 18406 | 0.26 | 0.89 | 3.30 |  |
| 2007 | 19716 | 0.26 | 0.89 | 3.36 |  |
| 2008 | 18422 | 0.25 | 0.87 | 3.33 |  |
| 2009 | 18643 | 0.25 | 0.89 | 3.27 |  |
| **Day of week** |  |  |  |  |  |
| Sunday | 15476 | 0.22 | 0.92 | 3.84 | <0.0001 |
| Monday | 24394 | 0.23 | 0.79 | 2.88 |  |
| Tuesday | 25586 | 0.23 | 0.78 | 2.89 |  |
| Wednesday | 26087 | 0.24 | 0.80 | 2.97 |  |
| Thursday | 26420 | 0.24 | 0.80 | 2.99 |  |
| Friday | 29974 | 0.26 | 0.86 | 3.13 |  |
| Saturday | 23564 | 0.26 | 1.03 | 4.08 |  |

*P value of ANOVA F-test

Table A2 Median crash distance among cyclists in England 2000-2009

| **Characteristic** | **n** | **25th** | **50th** | **75th** | **Pvalue*** |
| --- | --- | --- | --- | --- | --- |
| **Age** | | | | | |
| 0-5 | 793 | 0.06 | 0.14 | 0.45 | <0.0001 |
| 6-10 | 6646 | 0.09 | 0.20 | 0.50 |  |
| 11-15 | 16749 | 0.29 | 0.69 | 1.40 |  |
| 16-20 | 10543 | 0.62 | 1.39 | 2.78 |  |
| 21-25 | 9079 | 0.82 | 1.81 | 3.78 |  |
| 26-30 | 10838 | 1.01 | 2.16 | 4.46 |  |
| 31-35 | 10336 | 1.02 | 2.27 | 4.92 |  |
| 36-40 | 9631 | 1.02 | 2.36 | 5.29 |  |
| 41-45 | 6408 | 1.02 | 2.33 | 5.28 |  |
| 46-50 | 7483 | 0.90 | 2.13 | 4.82 |  |
| 51-55 | 4497 | 0.88 | 1.97 | 4.43 |  |
| 56-60 | 3495 | 0.74 | 1.76 | 4.02 |  |
| 61-65 | 2147 | 0.71 | 1.69 | 4.11 |  |
| 66-70 | 1324 | 0.59 | 1.36 | 3.72 |  |
| 71+ | 2023 | 0.46 | 0.98 | 2.42 |  |
| **Sex** | | | | | |
| Male | 82561 | 0.58 | 1.54 | 3.71 | <0.0001 |
| Female | 21146 | 0.50 | 1.23 | 2.72 |  |
| **Severity** | | | | | |
| Fatal | 651 | 0.77 | 2.15 | 5.30 | <0.0001 |
| Serious | 14575 | 0.59 | 1.57 | 3.89 |  |
| Slight | 88493 | 0.56 | 1.44 | 3.41 |  |
| **IMD of home residence** | | | | | |
| 1 | 8379 | 0.71 | 1.81 | 4.99 | <0.0001 |
| 2 | 8242 | 0.70 | 1.82 | 4.70 |  |
| 3 | 8487 | 0.65 | 1.72 | 4.32 |  |
| 4 | 8862 | 0.63 | 1.62 | 4.01 |  |
| 5 | 9306 | 0.62 | 1.57 | 3.86 |  |
| 6 | 10365 | 0.58 | 1.47 | 3.40 |  |
| 7 | 11393 | 0.54 | 1.36 | 3.19 |  |
| 8 | 12234 | 0.53 | 1.41 | 3.25 |  |
| 9 | 13233 | 0.51 | 1.34 | 3.04 |  |
| 10 | 13218 | 0.38 | 1.09 | 2.49 |  |
| **Urban/Rural status** | | | | | |
| Urban | 87771 | 0.53 | 1.36 | 3.11 | <0.0001 |
| Rural | 15907 | 0.78 | 2.47 | 6.38 |  |

| **Characteristic** | **n** | **25th** | **50th** | **75th** | **Pvalue*** |
| --- | --- | --- | --- | --- | --- |
| **Government Office Region** | | | | | |
| North East | 3467 | 0.33 | 1.07 | 2.95 | <0.0001 |
| North West | 13685 | 0.43 | 1.28 | 3.16 |  |
| Yorkshire and the Humber | 10227 | 0.48 | 1.29 | 3.06 |  |
| East Midlands | 6164 | 0.48 | 1.26 | 2.98 |  |
| West Midlands | 10473 | 0.45 | 1.20 | 2.79 |  |
| East of England | 11929 | 0.50 | 1.20 | 2.66 |  |
| London | 21512 | 0.99 | 2.46 | 5.19 |  |
| South East | 17906 | 0.57 | 1.35 | 3.20 |  |
| South West | 8181 | 0.59 | 1.44 | 3.06 |  |
| **Year** |  |  |  |  |  |
| 2000 | 8854 | 0.49 | 1.31 | 3.06 | <0.0001 |
| 2001 | 8925 | 0.51 | 1.35 | 3.17 |  |
| 2002 | 8185 | 0.52 | 1.36 | 3.18 |  |
| 2003 | 8337 | 0.51 | 1.33 | 3.26 |  |
| 2004 | 9165 | 0.49 | 1.35 | 3.14 |  |
| 2005 | 11098 | 0.53 | 1.41 | 3.36 |  |
| 2006 | 11364 | 0.60 | 1.50 | 3.50 |  |
| 2007 | 12299 | 0.62 | 1.54 | 3.63 |  |
| 2008 | 12079 | 0.63 | 1.62 | 3.86 |  |
| 2009 | 13413 | 0.67 | 1.74 | 4.18 |  |
| **Day of week** |  |  |  |  |  |
| Sunday | 8611 | 0.45 | 1.38 | 4.26 | <0.0001 |
| Monday | 15696 | 0.56 | 1.45 | 3.36 |  |
| Tuesday | 17763 | 0.62 | 1.55 | 3.58 |  |
| Wednesday | 17716 | 0.60 | 1.52 | 3.54 |  |
| Thursday | 17380 | 0.60 | 1.50 | 3.45 |  |
| Friday | 16193 | 0.56 | 1.44 | 3.33 |  |
| Saturday | 10360 | 0.44 | 1.27 | 3.33 |  |

*P value of ANOVA F-test

Table A3 Median crash distance among powered two-wheelers in England 2000-2009

| **Characteristic** | **n** | **25th** | **50th** | **75th** | **Pvalue*** |
| --- | --- | --- | --- | --- | --- |
| **Age** | | | | | |
| 0-5 | 34 | 0.14 | 1.09 | 4.37 | <0.0001 |
| 6-10 | 196 | 0.67 | 2.75 | 8.93 |  |
| 11-15 | 1572 | 0.47 | 1.18 | 3.37 |  |
| 16-20 | 43548 | 1.02 | 2.54 | 5.66 |  |
| 21-25 | 19364 | 1.57 | 4.04 | 9.81 |  |
| 26-30 | 19330 | 1.96 | 5.05 | 11.77 |  |
| 31-35 | 19249 | 2.18 | 5.69 | 13.52 |  |
| 36-40 | 18522 | 2.28 | 6.13 | 14.91 |  |
| 41-45 | 12201 | 2.35 | 6.38 | 16.67 |  |
| 46-50 | 12466 | 2.31 | 6.54 | 17.14 |  |
| 51-55 | 6443 | 2.36 | 6.61 | 17.43 |  |
| 56-60 | 3980 | 2.16 | 6.17 | 16.45 |  |
| 61-65 | 2006 | 1.68 | 4.65 | 13.87 |  |
| 66-70 | 874 | 1.68 | 5.00 | 16.11 |  |
| 71+ | 794 | 1.09 | 2.75 | 8.52 |  |
| **Sex** | | | | | |
| Male | 145052 | 1.60 | 4.33 | 10.86 | 0.1972 |
| Female | 17545 | 1.57 | 4.11 | 10.35 |  |
| **Severity** | | | | | |
| Fatal | 3264 | 2.58 | 7.23 | 18.51 | <0.0001 |
| Serious | 38291 | 1.73 | 4.90 | 12.96 |  |
| Slight | 121069 | 1.54 | 4.09 | 10.09 |  |
| **IMD of home residence** | | | | | |
| 1 | 13850 | 2.17 | 6.12 | 15.67 | <0.0001 |
| 2 | 15190 | 2.15 | 5.86 | 14.42 |  |
| 3 | 15885 | 2.01 | 5.44 | 13.59 |  |
| 4 | 16229 | 1.89 | 5.07 | 12.13 |  |
| 5 | 16333 | 1.74 | 4.75 | 11.65 |  |
| 6 | 17069 | 1.61 | 4.29 | 10.76 |  |
| 7 | 17832 | 1.50 | 3.94 | 9.75 |  |
| 8 | 18540 | 1.37 | 3.58 | 8.80 |  |
| 9 | 17554 | 1.23 | 3.24 | 7.77 |  |
| 10 | 14142 | 1.08 | 2.78 | 6.75 |  |
| **Urban/Rural status** | | | | | |
| Urban | 111531 | 1.25 | 3.23 | 7.86 | <0.0001 |
| Rural | 51048 | 3.28 | 8.10 | 19.83 |  |

| **Characteristic** | **n** | **25th** | **50th** | **75th** | **Pvalue*** |
| --- | --- | --- | --- | --- | --- |
| **Government Office Region** | | | | | |
| North East | 4361 | 1.26 | 3.80 | 10.22 | <0.0001 |
| North West | 16936 | 1.34 | 3.61 | 9.08 |  |
| Yorkshire and the Humber | 13458 | 1.44 | 3.94 | 10.55 |  |
| East Midlands | 11891 | 1.62 | 4.66 | 12.67 |  |
| West Midlands | 15674 | 1.34 | 3.50 | 8.68 |  |
| East of England | 19984 | 1.59 | 4.42 | 11.89 |  |
| London | 39719 | 2.04 | 5.05 | 10.94 |  |
| South East | 27471 | 1.57 | 4.36 | 12.03 |  |
| South West | 12862 | 1.49 | 3.97 | 10.24 |  |
| **Year** |  |  |  |  |  |
| 2000 | 13687 | 1.72 | 4.56 | 11.49 | <0.0001 |
| 2001 | 15352 | 1.71 | 4.39 | 10.91 |  |
| 2002 | 14808 | 1.62 | 4.46 | 10.79 |  |
| 2003 | 15207 | 1.62 | 4.40 | 10.99 |  |
| 2004 | 15665 | 1.55 | 4.06 | 10.26 |  |
| 2005 | 18057 | 1.48 | 4.13 | 10.60 |  |
| 2006 | 17396 | 1.57 | 4.23 | 10.58 |  |
| 2007 | 18830 | 1.56 | 4.27 | 10.94 |  |
| 2008 | 16820 | 1.58 | 4.28 | 10.55 |  |
| 2009 | 16802 | 1.64 | 4.38 | 11.16 |  |
| **Day of week** |  |  |  |  |  |
| Sunday | 19795 | 1.86 | 5.92 | 18.19 | <0.0001 |
| Monday | 22838 | 1.55 | 4.09 | 9.98 |  |
| Tuesday | 24120 | 1.58 | 4.08 | 9.63 |  |
| Wednesday | 25042 | 1.61 | 4.27 | 10.09 |  |
| Thursday | 23989 | 1.55 | 4.01 | 9.55 |  |
| Friday | 25799 | 1.56 | 4.20 | 10.42 |  |
| Saturday | 21041 | 1.53 | 4.29 | 11.53 |  |

*P value of ANOVA F-test

Table A4 Median crash distance among car occupants in England 2000-2009

| **Characteristic** | **n** | **25th** | **50th** | **75th** | **Pvalue*** |
| --- | --- | --- | --- | --- | --- |
| **Age** | | | | | |
| 0-5 | 15839 | 1.30 | 3.60 | 10.21 | <0.0001 |
| 6-10 | 21952 | 1.16 | 3.27 | 9.77 |  |
| 11-15 | 25508 | 1.32 | 3.56 | 9.85 |  |
| 16-20 | 161776 | 1.72 | 4.14 | 9.66 |  |
| 21-25 | 147959 | 1.79 | 4.76 | 12.66 |  |
| 26-30 | 118977 | 1.81 | 4.94 | 13.44 |  |
| 31-35 | 106664 | 1.68 | 4.63 | 12.80 |  |
| 36-40 | 100174 | 1.63 | 4.50 | 12.20 |  |
| 41-45 | 67371 | 1.72 | 4.65 | 12.60 |  |
| 46-50 | 82631 | 1.76 | 4.85 | 13.25 |  |
| 51-55 | 53711 | 1.87 | 5.10 | 14.09 |  |
| 56-60 | 43912 | 1.89 | 5.14 | 14.62 |  |
| 61-65 | 30193 | 1.76 | 5.04 | 14.71 |  |
| 66-70 | 22203 | 1.60 | 4.62 | 13.74 |  |
| 71+ | 45027 | 1.28 | 3.90 | 11.53 |  |
| **Sex** | | | | | |
| Male | 527816 | 1.82 | 4.88 | 13.23 | <0.0001 |
| Female | 529966 | 1.57 | 4.22 | 11.22 |  |
| **Severity** | | | | | |
| Fatal | 7658 | 2.69 | 6.60 | 16.84 | <0.0001 |
| Serious | 73585 | 2.08 | 5.34 | 13.82 |  |
| Slight | 976817 | 1.65 | 4.46 | 12.04 |  |
| **IMD of home residence** | | | | | |
| 1 | 97941 | 2.28 | 6.09 | 15.51 | <0.0001 |
| 2 | 104847 | 2.17 | 5.72 | 14.55 |  |
| 3 | 107115 | 2.10 | 5.53 | 14.21 |  |
| 4 | 107671 | 2.00 | 5.28 | 13.60 |  |
| 5 | 105391 | 1.91 | 5.03 | 13.20 |  |
| 6 | 106020 | 1.71 | 4.68 | 12.53 |  |
| 7 | 102776 | 1.59 | 4.23 | 11.70 |  |
| 8 | 105633 | 1.42 | 3.79 | 10.25 |  |
| 9 | 106520 | 1.29 | 3.40 | 9.27 |  |
| 10 | 114146 | 1.13 | 2.92 | 7.63 |  |
| **Urban/Rural status** | | | | | |
| Urban | 590051 | 1.09 | 2.80 | 6.92 | <0.0001 |
| Rural | 467630 | 3.43 | 8.26 | 21.70 |  |

| **Characteristic** | **n** | **25th** | **50th** | **75th** | **Pvalue*** |
| --- | --- | --- | --- | --- | --- |
| **Government Office Region** | | | | | |
| North East | 45988 | 1.64 | 4.26 | 10.29 | <0.0001 |
| North West | 167362 | 1.40 | 3.65 | 9.39 |  |
| Yorkshire and the Humber | 116130 | 1.64 | 4.26 | 11.29 |  |
| East Midlands | 83029 | 1.99 | 5.54 | 15.85 |  |
| West Midlands | 141184 | 1.48 | 3.80 | 9.57 |  |
| East of England | 137765 | 2.06 | 6.04 | 16.80 |  |
| London | 114885 | 1.39 | 3.58 | 8.38 |  |
| South East | 170879 | 1.99 | 5.59 | 15.80 |  |
| South West | 79437 | 2.08 | 5.81 | 16.89 |  |
| **Year** |  |  |  |  |  |
| 2000 | 94471 | 1.63 | 4.37 | 11.65 | <0.0001 |
| 2001 | 101672 | 1.61 | 4.40 | 11.81 |  |
| 2002 | 98357 | 1.64 | 4.41 | 11.88 |  |
| 2003 | 93464 | 1.66 | 4.44 | 12.11 |  |
| 2004 | 105501 | 1.66 | 4.47 | 11.96 |  |
| 2005 | 118843 | 1.70 | 4.58 | 12.33 |  |
| 2006 | 116669 | 1.71 | 4.59 | 12.33 |  |
| 2007 | 118173 | 1.77 | 4.71 | 12.76 |  |
| 2008 | 104841 | 1.71 | 4.60 | 12.23 |  |
| 2009 | 106069 | 1.74 | 4.71 | 12.71 |  |
| **Day of week** |  |  |  |  |  |
| Sunday | 137236 | 1.83 | 5.08 | 14.80 | <0.0001 |
| Monday | 145490 | 1.66 | 4.46 | 11.80 |  |
| Tuesday | 146835 | 1.64 | 4.36 | 11.26 |  |
| Wednesday | 148640 | 1.66 | 4.42 | 11.47 |  |
| Thursday | 149561 | 1.65 | 4.39 | 11.54 |  |
| Friday | 172269 | 1.67 | 4.55 | 12.53 |  |
| Saturday | 158029 | 1.71 | 4.57 | 12.66 |  |

*P value of ANOVA F-test
